# SECS, drugs, and Rac1&Rho: regulation of EnNaC in vascular endothelial cells

**DOI:** 10.1007/s00424-025-03093-5

**Published:** 2025-05-22

**Authors:** Benedikt Fels, Felix Fischer, Lisbeth Herrnboeck, David Beckers, Leon Niedzielski, Paul Roche, Alexandra Straeter, Ioana Alesutan, Johanna-Theres Borutta, Frederic Jaisser, Olivier Staub, Jakob Voelkl, Kristina Kusche-Vihrog

**Affiliations:** 1 Institute of Physiology, University of Lübeck, Ratzeburger Str. 160, 23562 Lübeck, Germany; 2 Institute of Physiology II, University of Münster, Münster, Germany; 3 INSERM, UMRS 1166, ICAN, Sorbonne Université, Paris, France; 4 Université de Lorraine, INSERM Centre d’Investigations Cliniques-Plurithématique 1433, UMR 1116, CHRU de Nancy, Nancy, France; 5 Department of Biomedical Sciences, University of Lausanne, Lausanne, Switzerland; 6 Institute for Physiology and Pathophysiology, Johannes Kepler University Linz, Linz, Austria; 7 Department of Nephrology and Medical Intensive Care, Charité-Universitätsmedizin Berlin, Corporate Member of Freie Universität Berlin and Humboldt Universität zu Berlin, Berlin, Germany; 8 DZHK (German Centre for Cardiovascular Research), Partner Site Berlin, Berlin, Germany; 9 DZHK (German Research Centre for Cardiovascular Research), Partner Site Hamburg/Luebeck/Kiel, Luebeck, Germany

**Keywords:** EnNaC, Endothelial nanomechanics, Non-genomic aldosterone effects, AFM

## Abstract

The endothelial ENaC (EnNaC) is mainly responsible for maintaining the mechanical properties of the endothelial cell surface, the sensitivity to the shear forces of the streaming blood and thus for vascular function. The correlation between EnNaC surface expression, the dynamics of the actin cortex, the mechanical stiffness, and nitric oxide release indicates a close structure–function relationship. Mechanical flexibility of the endothelial surface has been associated with proper vascular function, while chronic stiffening leads to endothelial dysfunction and the so-called ‘stiff endothelial cell syndrome’ (SECS). With the help of atomic force microscopy (AFM)–based nanoindentation and immunofluorescence staining in vitro and ex vivo, we investigated the underlying cellular mechanisms and signalling pathways of EnNaC-dependent endothelial behaviour. We were able to show that the interaction between EnNaC and the cortical cytoskeleton is mediated by the small GTPases RhoA, Rac1, and the Arp2/3 complex. The functional inhibition of EnNaC by the drugs amiloride and benzamil led to membrane removal of the channel within minutes. Furthermore, we could observe an involvement of mineralocorticoid receptor, SGK1 and Nedd4-2 in regulation of endothelial cell stiffness. Our study contributes further insights on complex regulation of EnNaC and elucidates its interaction with the actin cytoskeleton, which could be central to its role as a key regulator of vascular function in health and disease.

**Supplementary Information:**

The online version contains supplementary material available at 10.1007/s00424-025-03093-5.

## Introduction

The epithelial Na^+^ channel (ENaC) is known as a nonvoltage‐gated and amiloride‐sensitive sodium channel [47, 76]. ENaC is expressed at the apical membrane of epithelial cells and functions to transport Na^+^ down its electrochemical gradient through the cell. Canonical ENaC comprises of α, β, and γ subunits [65], whereas all three subunits are required to form the functional channel complex [10, 29, 36]. A fourth subunit, δ‐ENaC, has also been reported in specific tissues that can assemble with β‐ and γ‐ENaC and form a channel with unique electrophysiological properties compared to the ‘classical’ ENaC [5, 31]. At the cellular level, ENaC regulation involves a complex interplay of extracellular factors and intracellular signal transduction pathways. In epithelial tissues, the regulation of ENaC expression, membrane insertion/internalization, and activation are studied in detail (for review see [49, 76, 107]. ENaC is regulated by hormones (e.g., mineralocorticoid/aldosterone, vasopressin, glucocorticoids, insulin), ions (e.g., Na^+^), phospholipids (e.g., PIP2 and PIP3, involving MARCKS proteins), several proteins (e.g., Nedd4-2, SGK1, proteases, and kinases [1, 4, 17, 75, 105]), factor VII activating protease (FSAP) [3] by posttranslational modifications (e.g., ubiquitylation, phosphorylation, acetylation, and palmitoylation), and stretch activation [8, 22, 50, 51, 86]. Specific drugs can be used in vitro and in vivo to inhibit ENaC function (e.g., amiloride, benzamil) [60].

ENaC also plays an important role in non-renal tissues such as the brain, vascular endothelium, and vascular smooth muscle cells (VSMC) [20, 41, 56, 69, 71, 73]. The expression of all ENaC subunits was described in endothelial cells [55] and VSMC [19]. Dysbalance in the expression of endothelial ENaC subunits and mutations were shown to alter channel function and lead to differences in the responsiveness of the vascular endothelium and to pathophysiological conditions [7, 39, 63, 64]. In this context, Paudel et al. reported the expression of the δ subunit in human arteries having an important role in vascular function and the development of arterial hypertension [68]. In contrast to the epithelial ENaC, endothelial ENaC (here termed EnNaC) [100] has been attributed to function as (i) vascular mechanosensor [14, 51], (ii) regulator of vessel tone [68, 91], and (iii) as membrane anchored structural protein [37, 48]. Thus, instead of acting solely as an Na^+^ transporter, it seems that one of the most important features of EnNaC is the ability to maintain the mechanical properties and function of the endothelial cell surface as this property modulates the bioavailability of nitric oxide (NO) and consequently relaxation of VSMC [14, 24, 35, 39, 101]. Of note, a direct correlation between EnNaC surface expression and stiffness could be demonstrated in vascular endothelial cells [40], indicating a strong structure–function relationship of the endothelial version of the channel. Thus, a continuous, EnNaC-dependent change between the ‘soft’ and ‘stiff’ condition of the endothelial surface and appropriate NO release [14, 26–28] is crucial for proper vascular function and behaviour. In case this EnNaC-mediated mechanism is diminished, a pathophysiological condition defined as ‘stiff endothelial cell syndrome’ (SECS) could occur, leading to chronic endothelial dysfunction and inflammatory processes [57].

Aldosterone and high physiological extracellular Na^+^ concentrations, for example, induce an EnNaC-dependent stiffening of the endothelial surface and consequently a reduction of the NO release [52, 79], while functional inhibition of EnNaC with amiloride leads to channel retrieval [55], softens the endothelial cell surface, increases the NO release [14, 39, 91], and reduces the arterial stiffness [61]. For the vascular reactivity of EnNaC, its connection to the actin molecules of the endothelial cortex, located 150–200 nm beneath the plasma membrane, is of great importance. Moreover, the endothelial NO-synthase (eNOS) is co-localized with cortical F-actin, and it could be shown that polymerization of the actin cytoskeleton (shift from G- to F-actin) decreases eNOS activity/NO release [89] and also vice versa [14, 25, 26].

Similar to ENaC, at the cellular level, the expression and membrane insertion of EnNaC are regulated by aldosterone and the mineralocorticoid receptor (MR). This can be attributed to genomic pathways where aldosterone binding to cytosolic MR results in translocation of the MR into the nucleus, where it acts as a transcription factor. Although most of the aldosterone actions can be attributed to its genomic activity, it can also operate by yet unresolved non-genomic and rapid mechanisms [11, 13, 80–82, 92]. In this context, it was shown that shear stress induces a rapid, non-genomic membrane insertion [14].

However, the underlying mechanisms of all these observations are not clear yet. To investigate the cellular mechanisms of EnNaC-dependent alterations in the mechanical properties and function of endothelial cells, we employed atomic force microscopy (AFM)–based nanoindentation measurements to quantify the stiffness of the endothelial surface as a function of actin-based cytoskeletal dynamics. Therefore, we mainly focus on mediators such as small GTPases, kinases, and specific EnNaC inhibitors and the role of the cytoskeleton in the modulation of EnNaC expression, membrane insertion/internalization, and the mechanical consequences for the vascular reactivity.

## Methods

### Cell and tissue culture

Cell culture conditions: Human endothelial EA.hy926 cells (kindly provided by Cora-Jean S. Edgell, University of North Carolina, Chapel Hill, NC, USA) were grown under standard cell culture conditions as described elsewhere [23]. Primary human endothelial cells from the umbilical cord vein (human umbilical vein endothelial cells; HUVEC) were isolated as described before in detail [95]. Umbilical cords were donated by patients giving birth in the Marien-Krankenhaus Luebeck as well as the University Medical Centre Schleswig–Holstein Campus Luebeck (approved by Local Ethics Committee Cases: 18–325 and 2023-520_1). Cells were cultured in standard cell culture media (EA.hy926: DMEM + foetal calf serum 10% + penicillin/streptomycin 1%, all from Gibco, Carlsbad, CA, USA; HUVEC: Medium 199 supplemented with foetal calf serum 10% (FCS), penicillin/streptomycin 1% (100 U/mL, 100 mg/mL), large vessel endothelia supplement 1% (all from Gibco, Carlsbad, CA, USA), and heparin (5000 U/mL; Biochrom, Schaffhausen, Switzerland) at 37 °C, 21% O_2_, and 5% CO_2_. For experiments, cells were seeded on thin glass coverslips (Ø = 15 mm) and used after reaching confluence (48–72 h). Ex vivo murine aortic patches were cultured at 37 °C and 5% CO_2_ in minimal essential medium (MEM, containing 1% MEM vitamins, 1% NEAA (all from Gibco, Carlsbad, CA, USA), penicillin G (10,000 U/mL), and streptomycin (10,000 µg/mL, both Biochrom, Berlin, Germany).

*In vitro* cell culture stimulation: After reaching confluence, cells were treated with the following substances, depending on the experiments: 0.1–10 nM aldosterone, 100 nM spironolactone, 1 µM amiloride, 0.1 µM benzamil, 50 nM cytochalasin D (CytD), 10 µM CK548 (all from Sigma Aldrich, St. Louis, MO, USA), 10 µM LY294002, 0.5 µM jasplakinolide (Thermo Fisher Scientific), 10 µM chelerythrin chloride, 5 µg/mL brefeldin A (both Biomol, Hamburg, Germany), 50 µM NSC23766 (Tocris, Bristol, UK), 1 µg/mL CT04 (Cytoskeleton, Denver, CO, USA), and 30 µM Pitstop-2 (Abcam, Cambridge, UK). For all stimulations, the appropriate solvent was applied to the control group. Unless otherwise stated, all cells were cultured in medium containing 1 nM aldosterone prior to the experiment to mimic physiological conditions.

**Fig. 1:**
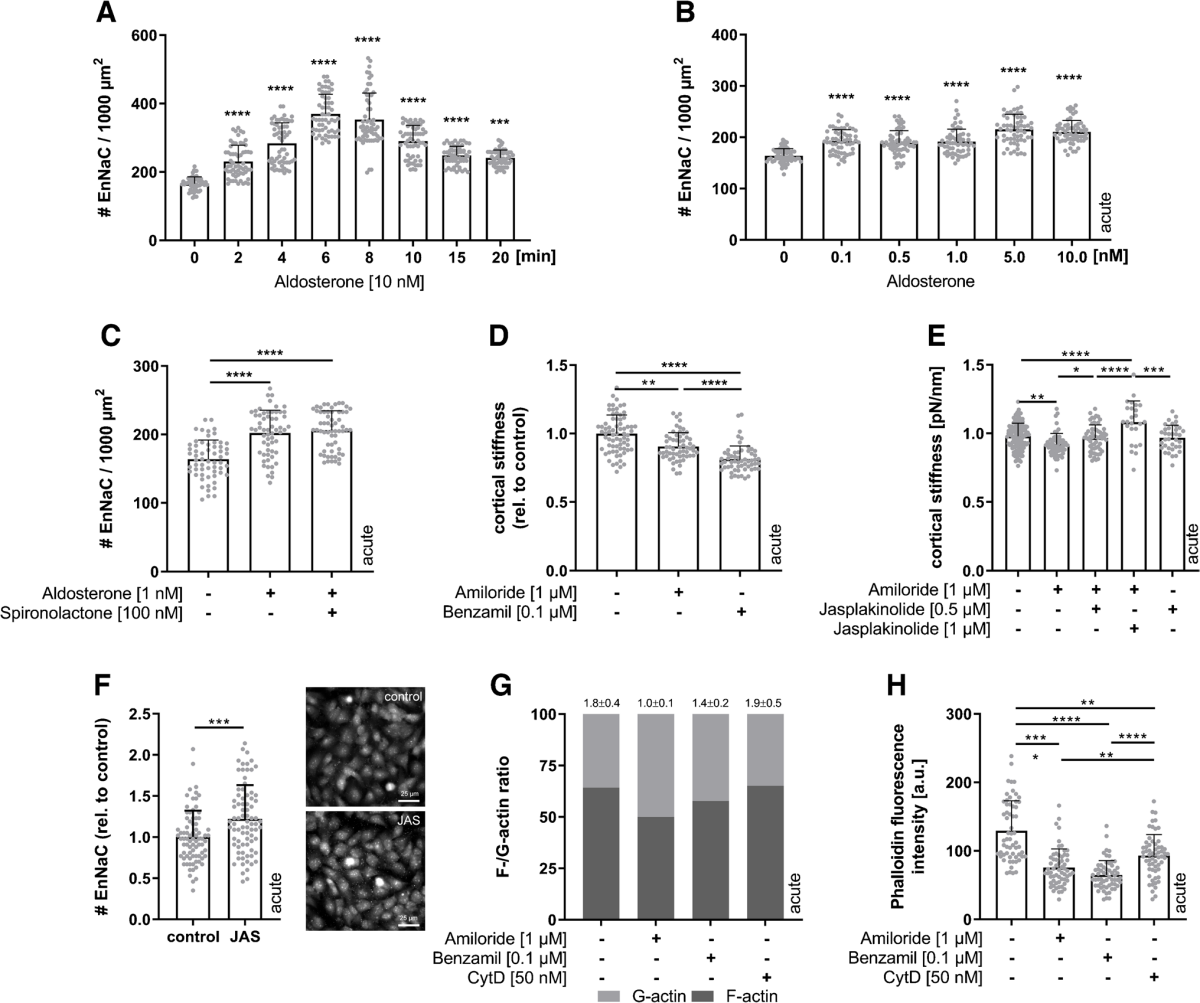
Rapid EnNaC membrane insertion and altered nanomechanics after aldosterone stimulation. **A** Time-series of 10 nM aldosterone application on EA.hy926 cells and quantification of EnNaC membrane abundance via Q-dot-based fluorescence staining. Already after 2 min of 10 nM aldosterone stimulation, a significant increase of the number of EnNaC within the membrane could be observed, reaching the highest channel density after 6 min (*N* = 3, *n* = 60; ****p < 0.0001 vs. control (0 min), Kruskal–Wallis test). **B** EnNaC density was analysed in a concentration series with 7 min stimulation in EA.hy926 cells, already showing an increase of EnNaC membrane abundance after stimulation with 0.1 nM aldosterone (*N* = 3, *n* = 60; ****p < 0.0001 vs. control (0 nM), Kruskal–Wallis test). **C** Short-term application (7 min) of 1 nM aldosterone was combined with the MR-antagonist spironolactone (100 nM), and EnNaC membrane abundance was analysed using EA.hy926 cells. Aldosterone increased the number of EnNaC within the membrane, whereas spironolactone did not additionally affect the EnNaC number (*N* = 3, *n* = 60; ****p < 0.0001 vs. control). **D** Functional EnNaC inhibition acutely changed cortical stiffness of EA.hy926 cells (stimulation < 2 min) after treatment with amiloride as well as benzamil measured by AFM-based nanoindentation (*N* = 3, *n* = 59–71; **p < 0.01, ****p < 0.0001 vs. control, Kruskal–Wallis test). **E** Stabilization of filamentous F-actin by Jasplakinolide abolished the Amiloride-induced softening of the actin cortex (EA.hy926, *N* = 5, *n* = 25–129, *p < 0.05, **p < 0.01, ****p < 0.0001 vs. control, ANOVA). **F** F-actin stabilization by Jasplakinolide (JAS) directly affected the number of EnNaC channels in the membrane (HUVECs, *N* = 4, *n* = 80, ***p < 0,001, Mann–Whitney test). **G** Alterations of F- to G-actin ratio in response to EnNaC inhibition were analysed in a western blot-based approach in EA.hy926. Amiloride and benzamil both led to a reduction of F-actin to G-actin proportion (*N* = 3, ns. vs. control). **H** Actin polymerization was analysed by phalloidin-TRITC fluorescence intensity analysis. Amiloride, benzamil, and the positive control, cytochalasin D (CytD), led to a significant reduction of phalloidin fluorescence (EA.hy926, *N* = 3, *n* = 60; *p < 0.05, ****p < 0.0001 vs. control, Kruskal–Wallis test)

For aldosterone-free conditions (see Fig. 1B + C), aldosterone was depleted from the cell culture media/foetal calf serum (FCS) using activated charcoal. The activated charcoal (20 g/L) was mixed with the required FCS and shaken for 24 h at 220 rpm at 37 °C. To subsequently remove the activated charcoal from the FCS, it was centrifuged three times at 1000 × g without braking and then sterilized by filtration. The FCS could now be used steroid-free as an additive to the cell culture medium.

### Animals and ethical approval for studies

Mouse models: All animal experiments were conducted according to the recommendations of the Guide for the Care and Use of Laboratory Animals of the NIH (National Academies Press) as well as of the German law for the welfare of animals and were approved by local authorities (agreement no. APAFIS#4488–20 16,010,614,517,136 v3, T026217). We used serum- and glucocorticoid-induced protein kinase 1 (SGK1)-KO mice and endothelial mineralocorticoid receptor (endoMR)-KO mice. The origin and generation of SGK1-KO and endoMR-KO mice have been described previously [103, 109]. The animals were genotyped by PCR using standard methods. Before the experiments, mice had free access to standard rodent diet and tap drinking water.

Dissection and preparation of mice tissue: Aortae of SGK1-KO and endoMR-KO mice were prepared as described before [94]. In brief, the thoracic aortae, from the heart to the diaphragm, were dissected from the mice (killed by cervical dislocation) and stored at 4 °C in a modified version (0.25 mM Ca^2+^) of solution 8 [106]. Cleaning of connective tissue under microscopic visualization and further preparations were conducted within 3–4 days [40].

### Immunofluorescence stainings of EnNaC and F-actin

EnNaC stainings: EnNaC abundance solely on the upper cell surface (facing the medium) of endothelial cells was detected and quantified via QD-based immunofluorescence. The cells were fixed in a non-permeabilizing manner and stained as described elsewhere [39, 55]. A primary polyclonal rabbit anti-αENaC antibody (1:250; Santa Cruz Biotechnology, Santa Cruz, CA, USA), a secondary Q-dot (QD)-labelled antibody (Q-dot 655 goat anti-rabbit IgG, 1:800; Invitrogen; Waltham, MA, USA), and DAPI (4′,6-diamidino-2-phenylindole; Invitrogen; Waltham, MA, USA) were used for staining after blocking with 10% normal goat serum for 30 min on ice. Negative controls were established by incubating cells solely with the secondary antibody. Staining was verified by epi-fluorescence microscopy (microscope: Leica DMI 6000B, Leica Microsystems; camera: CoolSNAPHQ, Photometrics). The QD-based immunofluorescence was quantified by counting QD/1000 µm^2^ of cell surface using the ImageJ software (Version 1.52a; National Institute of Health, Bethesda, MD, USA) or with the YT-Evaluation software (Version 2.1.12014; Synentec GmbH, Elmshorn, Germany). Images were taken in three different sections of the endothelial monolayer, and all images were analysed simultaneously in order to account for any variations in cell height. QD background levels (QD detected in negative controls) were subtracted from the results.

F-actin detection: Visualization of the cortical actin was done with phalloidin-TRITC (10 µg/mL, 1 h at RT, Sigma Aldrich, St. Louis, MO, USA) after cell fixation with 3.5% paraformaldehyde for 30 min on ice. Cells were permeabilized for 10 min with 0.1% Triton X-100 (Sigma Aldrich, St. Louis, MO, USA), and blocked with 10% normal goat serum for 30 min. After washing, the cells were mounted with Dako mounting media with DAPI (Agilent, Santa Clara, CA, USA). Fluorescence images were captured with an inverted confocal microscope (TCS SP8, Leica Microsystems, Wetzlar, Germany) equipped with a 63 × NA 1.4 objective, or with a Keyence Fluorescence Microscope BZ9000 (Keyence Corporation, Osaka, Japan; 60 × objective). The fluorescence intensity of phalloidin-TRITC-staining was analysed by using the ImageJ software (Version 1.52a; National Institute of Health, Bethesda, MD, USA).

### siNedd4-2 transfection and western blot detection

siNedd4-2 transfection: Knockdown of Nedd4-2 was done with a siRNA approach (siNedd4-2 kindly provided by Prof. O. Staub, Department of Pharmacology and Toxicology, Lausanne, Switzerland) using Lipofectamine RNAiMAX reagent (Invitrogen; Waltham, MA, USA) according to manufactures protocol. In brief, 150 pmol siRNA directed against Nedd4-2, 150 pmol MOCK siRNA (Ambion, Hamburg, Germany, # AM4611) and two times of 7.5 μL Lipofectamine 2000 were incubated each in 250 μL DMEM with 10% FCS without antibiotics for 5 min at room temperature. Each of the siRNA solutions was mixed with Lipofectamine and incubated for another 20 min. Afterward, siRNA and Lipofectamine solution were added to the cells. The medium was changed after 12 h. Forty-eight hours after transfection, the cells were washed and lysed.

Nedd4-2 expression: Western blot was carried out with 7.5% polyacrylamide gels and samples were run at 80 V for about 20 min in a Tris–Glycine-SDS buffer. Gel was loaded with 10 μg protein of murine kidney, 30 μg protein of HEK293 (both as positive control), 30 μg protein of transfected EA.hy926 with siRNA against Nedd4-2 or transfected with MOCK siRNA, and 30 μg protein of HUVEC lysate. For blotting, nitrocellulose membranes were used in a transfer buffer at 35 V at 4 °C. To block unspecific binding sites, the membranes were incubated in 5% milk for 45 min. Anti-Nedd4-2 antibody (kindly provided by Prof. O. Staub, Department of Pharmacology and Toxicology, Lausanne, Switzerland) was applied in a final concentration of 1:1000 in 1% non-fat milk at 4 °C overnight. The membrane was washed in TBST for 3 × 15 min. As a secondary antibody, goat anti-rabbit IgGs were diluted in 5% non-fat milk (1:20,000) for 2 h on the shaker. Again, the membrane was washed in TBST for 3 × 15 min. Protein bands were detected with Western Bright (advansta inc., San Jose, CA, USA).

F-G-actin ratio: For detection of filamentous (F −) and globular (G −) actin, the *G-Aktin/F-Aktin *In Vivo* Assay Kit* (Cytoskeleton, Denver, CO, USA) was used according to manufacturer protocol. In brief, EA.hy926 cells, grown in T25 culture flasks, were lysed with 300 µL lysis buffer (1% 100 mM ATP, 1% protease inhibitor cocktail). After ultra-centrifugation at 100,000 × g for 1 h, the F-actin fraction was pelleted, whereas G-actin remained in the supernatant. The F-actin fraction was depolymerized, and SDS buffer was added separately to each fraction. Standard SDS page and western blot were performed with 12% polyacrylamide gel, running at 80 mV for 1 h. Semi-dry blotting was done on nitrocellulose membrane with 2.5 mA/cm^2^ for 1 h. After blocking in 5% TBST-milk, kit primary antibody rabbit anti-actin was incubated 1:5000 for 1 h at RT and anti-rabbit HRP secondary antibody (1:10,000) for 1 h at RT. Chemiluminescence was detected after Luminol incubation with Chemidoc XRS Imaging System (Bio-Rad, Hercules, CA, USA) and analysed using the Quantity One software and ImageJ software (Version 1.52a; National Institute of Health, Bethesda, MD, USA).

### Atomic force microscopy measurements

AFM: The mechanical stiffness was determined using an AFM-based nanoindentation technique (MultiMode® SPM, Bruker, Karlsruhe, Germany), with a soft cantilever (spring constant 30 pN/nm; Novascan, Ames, IA, USA), and a polystyrene sphere as the tip (diameter: 10 μm) as described before [24]. A maximal loading force of 3.0 nN was applied. ECs were analysed in HEPES-buffer (HEPES: 4-(2-hydroxyethyl)−1-piperazineethanesulfonic acid; Buffer (in mM): 140 NaCl, 5 KCl, 1 CaCl_2_, 1 MgCl_2_, 5 glucose, 10 HEPES) supplemented with 1% FCS at 37 °C in a fluid chamber.

Ex vivo aortic patches: Harvest and preparation of aortae for ex vivo analysis by AFM to analyse the cortical stiffness of the mouse aortic endothelial cells were carried out as described before [40]. Briefly, aortae from SGK1- or endoMR-KO mice were freed from surrounding tissue. Small patches of the whole aorta (≈2 mm^2^) were attached on glass coverslips with Cell-Tak® (BD Biosciences, Bedford, MA, USA), with the endothelial surface facing upwards. After preparation, the aortic patches were cultured until the next day for AFM measurements in minimal essential medium (MEM; Invitrogen Corp., La Jolla, CA, USA) supplemented with 10% foetal calf serum (FCS; PAA Laboratories, Pasching, Austria), 1% MEM vitamins (Invitrogen), 1% MEM nonessential amino acids (Invitrogen), and 1% Penicillin/Streptomycin (100 U/mL; 100 mg/mL) under standardized cell culture conditions. Cortical stiffness values were calculated from force-distance curves with the Protein Unfolding and Nano-Indentation Analysis Software PUNIAS3D version 1.0 release 2.2 (Philippe Carl & Paul Dalhaimer, punias@free.fr).

### Statistical analysis

Statistical analysis was performed using the GraphPad Prism 8. Data are presented as average ± SD. Gaussian distribution was assessed using the D’Agostino and Pearson omnibus normality test. Outliers were identified using the ROUT outlier test based on the false discovery rate (FDR; *Q* value = 1%) before performing statistical analyses. For comparisons between two groups, Student’s *t*-test was applied for parametric data, and the Wilcoxon–Mann–Whitney test was used for non-parametric data, followed by post hoc analysis. For comparisons between three or more groups, a one-way ANOVA with Bonferroni’s correction for multiple comparisons was used for parametric data, while the Kruskal–Wallis test by ranks was used for non-parametric data, followed by post hoc analysis. Statistical significance was set at *p < 0.05, **p < 0.01, ***p < 0.0.001, and ****p < 0.0001.

## Results

### Aldosterone induces rapid EnNaC membrane insertion and cortical stiffening

Aldosterone is recognized as important regulator of EnNaC expression and membrane insertion [14, 49]. To study the underlying mechanisms of the EnNaC membrane insertion in response to short-term aldosterone application, confluent EA.hy926 cells were treated with 10 nM aldosterone in a time series from 2 to 20 min (Fig. 1A). Already after 2 min of aldosterone stimulation, a significant increase of EnNaC membrane abundance by + 39% compared to controls could be observed, followed by a time-dependent further increase with a peak value of + 113% after 6 min of aldosterone administration. Even significantly lower doses of aldosterone induced an increase of + 17% in channel density (Fig. 1B). Already 0.1 nM of aldosterone led to an increase after short-term application (7 min).

Of note, this rapid aldosterone-mediated regulation of EnNaC membrane insertion was independent of the MR activation. Aldosterone (7 min, 10 nM) led to an increase of EnNaC (+ 24% vs. control, Fig. 1C), which could not be prevented by the MR-antagonist spironolactone (7 min, 1 nM Aldosterone + 100 nM spironolactone; + 27% vs control).

Also, functional EnNaC inhibition rapidly affected the nanomechanical properties of the endothelial surface (Fig. 1D). AFM-based quantification of the cortical stiffness showed a decrease by − 10% after short-term application (< 2 min) of the functional EnNaC blocker amiloride (1 µM) and by − 19% using benzamil (0.1 µM).

Alterations of nanomechanical properties in response to acute EnNaC inhibition are mediated by changes in the level of actin polymerization. Stabilization of filamentous F-actin by 0.5 µM Jasplakinolide (JAS, a peptide of the marine sponge *Jaspis johnstoni*) abolished the amiloride (1 µM, 30 min) induced reduction of the cortical stiffness (Fig. 1E; control: 0.98 ± 0.09 pN/nm, amiloride: 0.92 ± 0.08 pN/nm, amiloride + JAS: 0.97 ± 0.09 pN/nm). Stimulation with higher JAS concentrations (1 µM) led to even stronger actin polymerization and an increase in cortical stiffness (1.08 ± 0.16 pN/Nm). Stabilization of the F-actin also directly affected EnNaC membrane abundance, leading to an increase of 22% of EnNaC channels compared to control (Fig. 1F).

This could be underpinned by the fact that functional EnNaC inhibition with amiloride or benzamil induced a shift from F-actin to G-actin. Under control conditions, the F-/G-actin ratio is 1.8 ± 0.4, indicating an F-actin proportion of 64.2% to the total actin (and 35.8% G-actin, Fig. 1G). The rapid decrease of cortical stiffness after functional EnNaC inhibition (30 min; Fig. 1D) was accompanied by a balancing of F-/G-actin ratio to 50% filamentous and 50% globular actin (amiloride) and to 57% F- and 43% G-actin (benzamil). In contrast, the actin-depolymerizing agent cytochalasin D (CytD) did not change the F-/G-actin ratio compared to control conditions (65% F- and 35% G-actin).

Analysis of phalloidin-TRITC stained cortical F-actin fluorescence intensity confirmed these results and showed a reduction of phalloidin fluorescence by − 42% and 50% after short-term functional EnNaC inhibition (30 min) compared to untreated controls (Fig. 1H, control 129.3 ± 43.8 a.u. vs. amiloride 75.7 ± 27.3 a.u., benzamil 64.8 ± 21.0 a.u.). The actin polymerization inhibitor CytD induced a F-actin reduction of −28% (CytD 93.7 ± 30.8 a.u.).

### EnNaC membrane insertion via the PI3 K/PKC signalling pathway

**Fig. 2:**
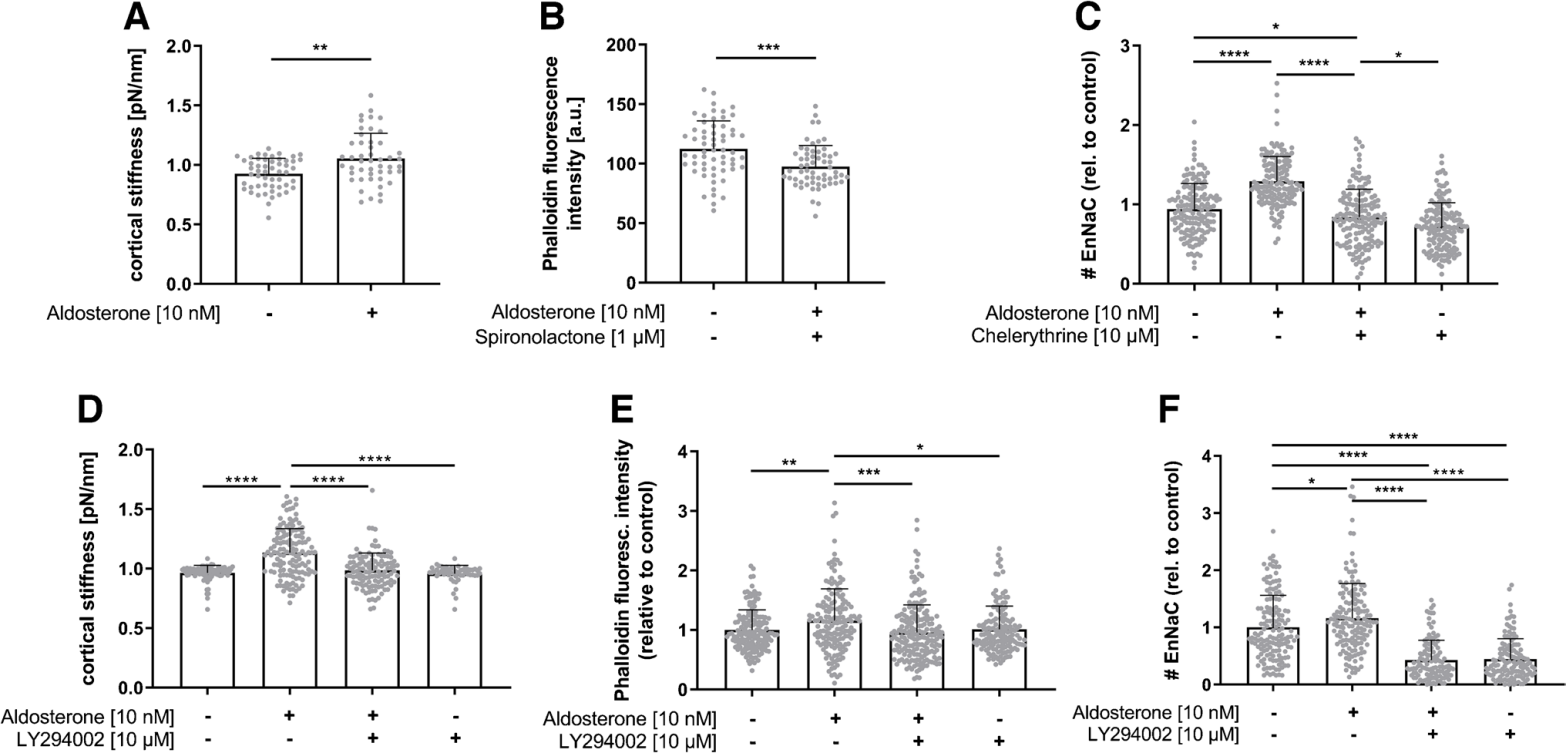
Non-genomic aldosterone response of EnNaC membrane insertion is mediated via the PI3 K/PKC signalling pathway. **A** Cortical nanomechanics were analysed after short-term stimulation with 10 nM aldosterone, leading to increased stiffness of the cortical actin (EA.hy926, *N* = 5, *n* = 125, **p < 0.01 vs. control, Mann–Whitney test). **B** Rapid aldosterone effects (30 min, 10 nM) on actin polymerization measured by phalloidin-TRITC fluorescence intensity were mitigated by co-stimulation with MR-antagonist spironolactone (EA.hy926, 1 µM, *N* = 3, *n* = 60, ***p < 0.001 vs. aldosterone; unpaired *t*-test). **C** Inhibition of PKC activity by chelerythrine (10 µM) prevented the rapid increase of EnNaC density after aldosterone stimulation (HUVECs, *N* = 3, *n* = 150, *p < 0.05, **p < 0.01, ****p < 0.0001 vs. control, Kruskal–Wallis test). **D** Aldosterone-induced alteration of cortical stiffness was decreased by pre-incubation of PI3 K pathway inhibitor LY294002 (HUVECs, 10 µM, *N* = 3, *n* = 114–132, ****p < 0.0001, unpaired *t*-test). **E** Actin polymerization was analysed by phalloidin-TRITC, confirming the rapid aldosterone-induced (10 min pre-incubation) increase of phalloidin fluorescence intensity. Incubation with LY234002 (10 µM) abolished this effect (HUVECs, *N* = 5, *n* = 360, *p < 0.05, **p < 0.01, ***p < 0.001 vs. control). **F** PI3 K inhibition via LY294002 also abolished the aldosterone-induced increase of EnNaC density back to the control level (HUVECs, *N* = 3, *n* = 150, *p < 0.05, ****p < 0.0001 vs. control, Kruskal–Wallis test)

To further elucidate the EnNaC function and signalling pathways in response to EnNaC activation/induction, we stimulated confluent endothelial cells for 30 min and analysed the nanomechanical properties of the cell surface (Fig. 2A). Application of aldosterone (10 nM) rapidly induces cortical stiffening (control: 0.93 ± 0.13 pN/nm vs. aldosterone 1.10 ± 0.21 pN/nm). Aldosterone-induced impact on cortical actin polymerization could be mitigated by co-stimulation with application of the MR-antagonist spironolactone (30 min, 1 µM, Fig. 2B; aldosterone 112.4 ± 23.4 a.u. vs aldosterone + spironolactone 97.5 ± 17.7 a.u.).

EnNaC membrane abundance in response to short-term aldosterone stimulation (+ 29% vs. control) could be altered by inhibition of the PKC pathway by chelerythrine (10 µM, 30 min) and lowered back to control levels (− 16% vs. control, Fig. 2C). Inhibition of the PI3 K pathway via LY294002 (pre-stimulation, 20 min, 10 µM) led to reduced cortical stiffness compared to aldosterone control group (Fig. 2D, aldosterone 1.13 ± 0.20 pN/Nm vs LY294002 0.99 ± 0.15 pN/nm). The increase of actin polymerization (+ 15% vs. control) after aldosterone stimulation (10 min, 10 nM) could be abolished by incubation with the PI3 K pathway inhibitor (10 µM), leading to −5% of phalloidin fluorescence intensity compared to control conditions (Fig. 2E). In addition, the aldosterone-dependent effects on EnNaC membrane insertion were abolished by PI3 K pathway inhibition (Fig. 2F: aldosterone + 15%, LY294002 − 70% EnNaC channel number).

### Small GTPases mediate dynamics of cortical actin

**Fig. 3:**
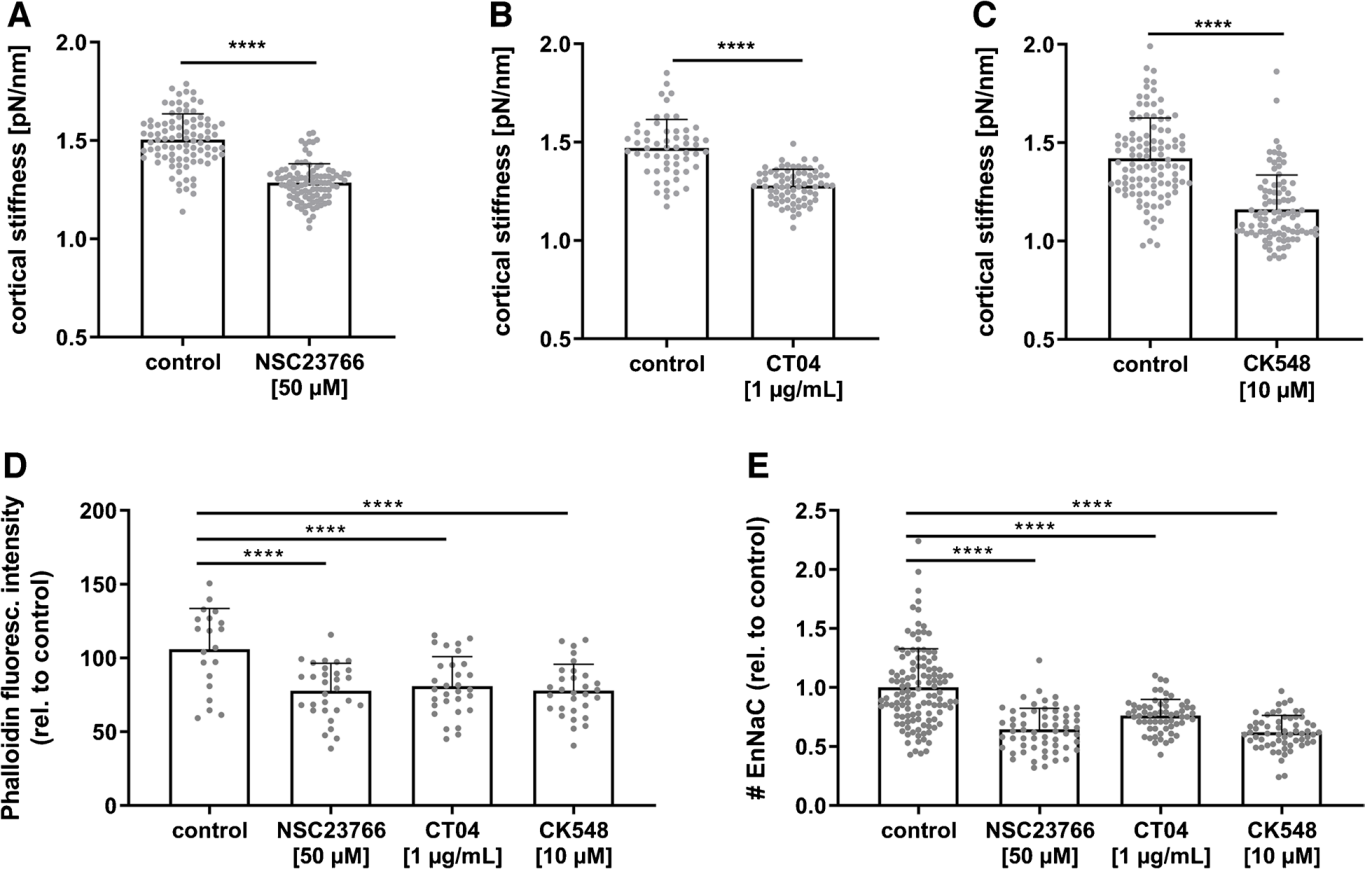
Inhibition of small GTPases led to reduced cortical stiffness and altered EnNaC membrane abundance. **A** NSC23766, an inhibitor of small GTPase Rac1, led to reduced cortical stiffness compared to the control group in EA.hy926 cells (10 µM, 24 h, *N* = 3, *n* = 92–97, ****p < 0.0001 vs. control, Mann–Whitney test). **B** Blockage of small GTPase RhoA by 1 mg/mL CT04 (24 h) reduced the stiffness of the endothelial actin cortex in EA.hy926 cells (*N* = 3, *n* = 57–74, ****p < 0.0001 vs. control, unpaired *t*-test). **C** Inhibition of Arp2/3 complex by 10 µM CK548 (24 h) initiated a reduced cortical stiffness (EA.hy926, *N* = 3, *n* = 93–108, ****p < 0.0001 vs. control, Mann–Whitney test). **D** Inhibition of Rac1, RhoA, Arp2/3 complex showed a reduced fluorescence intensity of phalloidin-TRITC stained F-actin in EA.hy926 cells (*N* = 3, *n* = 60–120, ****p < 0.0001 vs. control, ANOVA). **E** Q-Dot-based staining of EnNaC after inhibition of Rac1, RhoA, or Arp2/3 complex revealed a reduction of EnNaC membrane abundance in response to all 3 treatments (EA.hy926, *N* = 3–4, *n* = 60–120, ****p < 0.0001 vs. control, ANOVA)

The impact of the small GTPases Rac1 and RhoA on endothelial nanomechanics was analysed by selective GTPase inhibition and subsequent nanoindentation measurements. Inhibition of Rac1 (50 µM NSC23766, 24 h) led to a significant reduction of cortical stiffness by − 14.5 ± 0.7% (Fig. 3A; control 1.50 ± 0.13 pN/nm vs. NSC23766 1.29 ± 0.10 pN/Nm). Also, the inhibition of small GTPase RhoA (1 µg/mL CT04) induced a softening of the endothelial cortex (Fig. 3B; control 1.47 ± 0.15 pN/nm vs. CT04 1.28 ± 0.08 pN/nm). Stimulation with 10 µM CK548, inhibiting the actin-related proteins 2/3 (Arp2/3), led to a reduction of cortical stiffness as well (Fig. 3C; control 1.42 ± 0.20 pN/Nm vs. CK548 1.16 ± 0.18 pN/nm).

Softening of the endothelial cortex by blockage of small GTPase signalling could be confirmed with phalloidin-TRITC-based staining of cortical actin. Inhibition of Rac1, RhoA as well as Arp2/3 complex decreased phalloidin fluorescence intensity indicating reduced F-actin within the cortical actin network (Fig. 3D; Rac1 inhibition/NSC23766 − 22.2 ± 3.4%, RhoA inhibition/CT04 − 19.1 ± 3.7%, Arp2/3 inhibition/CK548 − 22.1 ± 3.3% vs. control).

Alteration of actin polymerization and cortical stiffness by small GTPase signalling cascades directly impacted on EnNaC membrane abundance. Inhibition of Rac1, RhoA, and Arp2/3 complex led to reduced number of Q-Dot labelled EnNaC channels in the membrane (Fig. 3E; NSC23766 −35%, CT04 − 24%, CK548 − 38%).

### Functional EnNaC inhibition leads to clathrin-mediated endocytosis

**Fig. 4:**
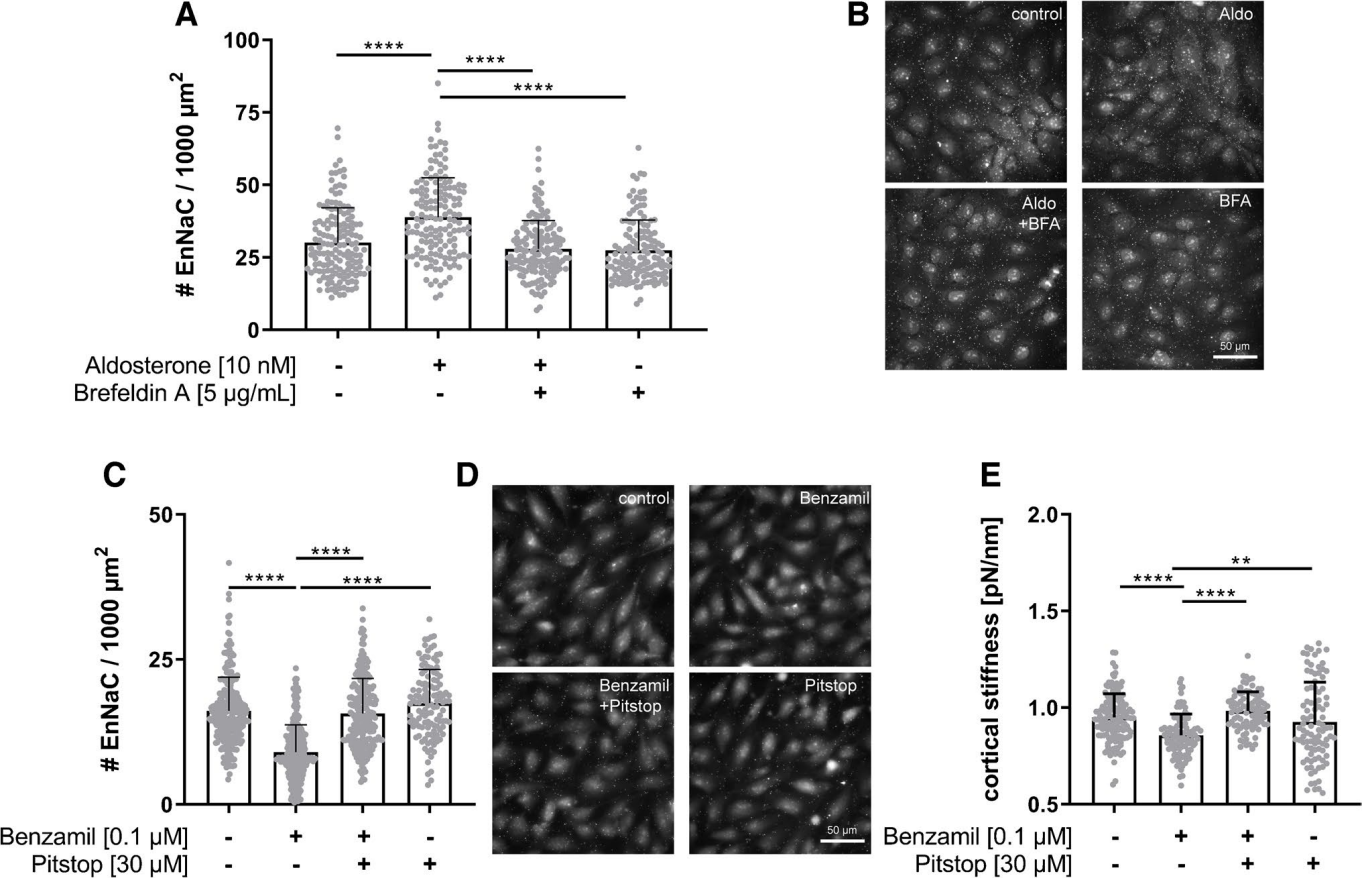
EnNaC inhibition led to Clathrin-mediated EnNaC endocytosis and prevents softening cortical actin layer. **A** Blockage of anterograde exocytotic vesicle transport via Brefeldin A (BFA, 5 µg/mL) abolished the rapid aldosterone-induced membrane insertion of EnNaC abundance in HUVECs (*N* = 3, *n* = 150, *p < 0.05, **p < 0.01, ****p < 0.0001 vs. control, Kruskal–Wallis test). **B** Representative Q-dot staining of EnNaC after stimulation with aldosterone and/or Brefeldin A (scale-bar: 50 µm). **C** Quantification of Q-dot stained EnNaC showed a Benzamil (0.1 µM)-induced reduction of the EnNaC number, whereas parallel Pitstop application prevented this clathrin-mediated EnNaC endocytosis (EA.hy926 cells, *N* = 3, *n* = 300, ****p < 0.0001 vs. control, Kruskal–Wallis test). **D** Representative Q-dot staining of EnNaC after stimulation with benzamil and co-treatment with clathrin-mediated endocytosis inhibitor Pitstop2 (scale bar: 50 µm). **E** EnNaC inhibition by 0.1 µM benzamil led to reduced cortical stiffness and could be prevented by co-stimulation with Clathrin-mediated endocytosis inhibitor Pitstop (EA.hy926 cells,30 µM, *N* = 3, *n* = 86–114, ****p < 0.0001 vs control, one-way ANOVA)

To test the involvement of vesicular transport of EnNaC between the endoplasmatic reticulum and the Golgi apparatus for EnNaC membrane insertion, we used Brefeldin A (BFA), a blocker of anterograde exocytotic transport. Preincubation with BFA 5 µg/mL, 30 min, prevented the acute effects of aldosterone treatment (10 min, 10 nM), resulting in a significantly lower amount of EnNaC in the plasma membrane supporting the role of vesicle transport and intracellular trafficking in ENaC membrane insertion (Fig. 4A).

Alteration of EnNaC function and abundance in response to EnNaC inhibition was also analysed using Pitstop2, a potent inhibitor of clathrin-dependent endocytosis. Quantification of EnNaC channel density showed a decrease in channel number after benzamil stimulation (Fig. 4B + C), and unaltered channel density after benzamil + Pitstop stimulation (30 µM, 30 min) compared to control conditions (Fig. 4C; control 15.3 ± 4.9, benzamil 8.9 ± 4.8, benzamil + pitstop 17.23 ± 6.9 #EnNaC per 1000 µm^2^ cell surface area).

Using AFM-based nanoindentation measurements, stimulation with the functional EnNaC inhibitor benzamil (0.1 µM) led to decreased cortical stiffness compared to control conditions (Fig. 4D; control 0.95 ± 0.12 pN/nm vs. benzamil 0.86 ± 0.11 pN/Nm). Inhibition of Clathrin-dependent endocytosis in addition to benzamil stimulation prevented alterations of cortical stiffness compared to the control level (benzamil + Pitstop 0.98 ± 0.10 pN/nm).

### Regulation of EnNaC membrane abundance in response to functional inhibition is mediated by Nedd4-2 and SGK1

**Fig. 5:**
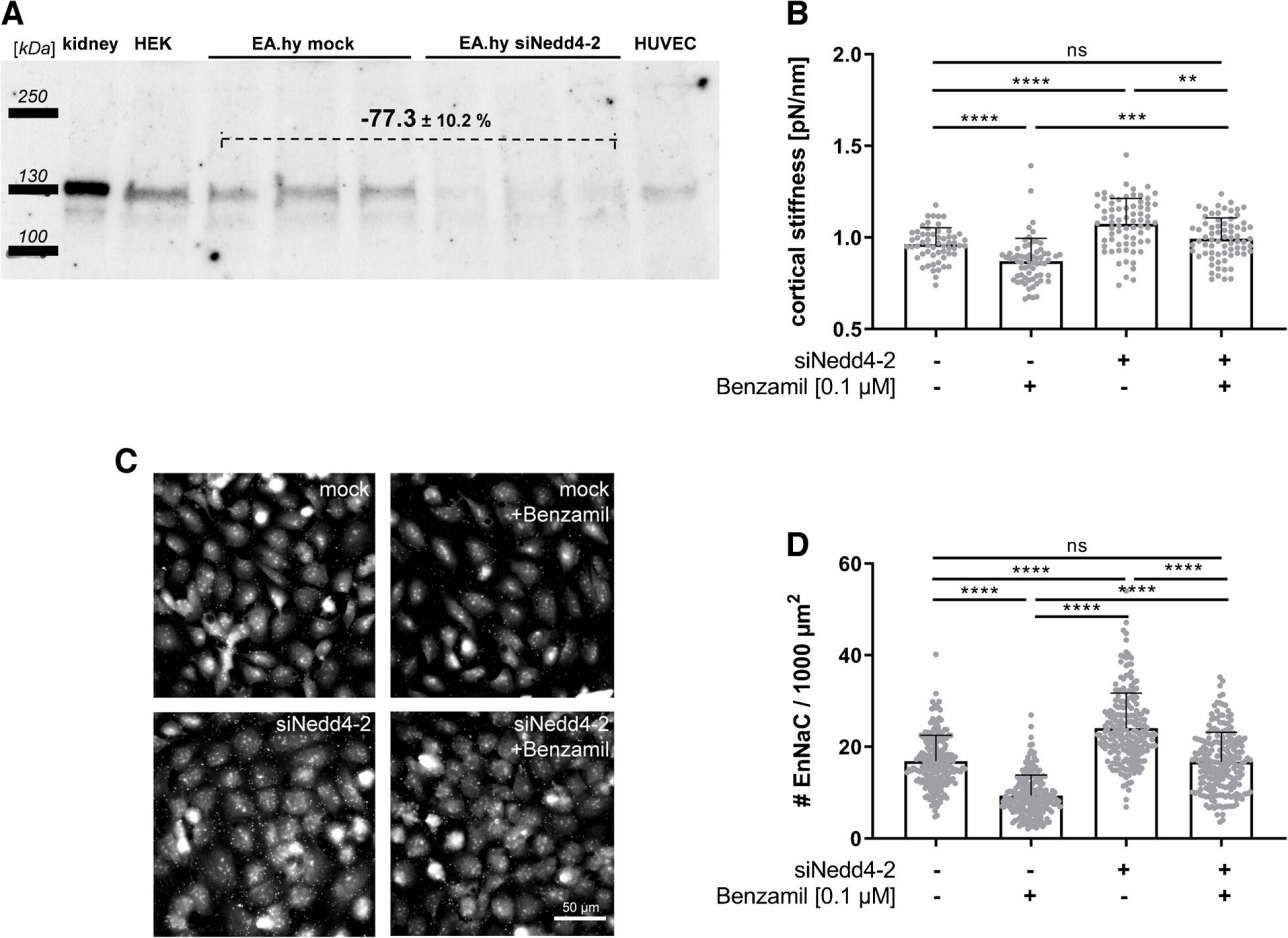
EnNaC-mediated alteration of cortical stiffness was mediated via Nedd4-2. (**A**) Western blot of siRNA-transfected EA.hy926 against Nedd4-2 showed a −77% reduced Nedd4-2 protein expression compared to mock-transfected cells. **(B)** Nanoindentation measurements with siNedd4-2-transfected EC vs. mock–transfected cells. Benzamil (0.1 µM) led to reduced cortical stiffness in mock-transfected EC compared to controls, whereas benzamil did not alter the cortical stiffness in siNedd4-2-transfected cells compared to controls (*N* = 3, *n* = 58–71, ** *p* < 0.01, **** *p* < 0.0001 vs. control, ANOVA). **(C)** Representative Q-dot staining of EnNaC in mock vs. siNedd4-2 transfected EA.hy926 cells with or without stimulation with benzamil (scale bar: 50 µm). **(D)** Quantification of Q-dot stained EnNaC in mock vs. siNedd4-2 transfected EA.hy926 cells showed a benzamil (0.1 µM)-induced reduction of the EnNaC number in mock transfected cells. Knockdown of Nedd4-2 lead to increased EnNaC membrane abundance (EA.hy926 cells, *N* = 3, *n* = 210, **** *p* < 0.0001, Kruskal–Wallis test)

ENaC membrane abundance is known to be regulated by endocytosis and exocytosis [9, 97, 102]. Nedd4-2 has been implicated to reduce ENaC membrane abundance by ubiquitination, an effect inhibited by SGK1-dependent phosphorylation [42, 43]. To study if the amiloride-dependent reduction of EnNaC numbers from the surface of endothelial cells [55] depends on disturbed internalization and turnover of the channel, endothelial cells were transfected with siRNA against ubiquitin ligase Nedd4-2. Western blot analysis confirmed a knock-down of Nedd4-2 by − 77.3 ± 10.2% compared to the mock-transfected control group (Fig. 5A). Nanoindentation measurements again confirmed a softening of the endothelial cortex by − 10% after functional inhibition of EnNaC with 0.1 µM benzamil (Fig. 5B; control 0.96 ± 0.09 pN/nm vs. benzamil 0.87 ± 0.12 pN/nm). In contrast, inhibition of EnNaC membrane removal via siNedd4-2 transfection showed increased cortical stiffness compared to controls (siNedd4-2 1.08 ± 0.19 pN/nm) and an attenuated benzamil-induced softening (siNedd4-2 + benzamil 0.99 ± 0.11 pN/nm). Quantification of Q-dot stained EnNaC showed reduced numbers of EnNac after benzamil treatment compared to untreated (mock-transfected) controls (see Fig. 5C + D). Knockdown of Nedd4-2 in EA.hy926 lead to increased EnNaC membrane abundance compared to mock-transfected controls. However, benzamil treatment of siNedd4-2 cells still reduced the EnNaC numbers, but to a lesser extent compared to the benzamil-treated control group, which can probably be explained by only partial knockdown of Nedd4-2 within this cells (refer to Fig. 5A).

**Fig. 6:**
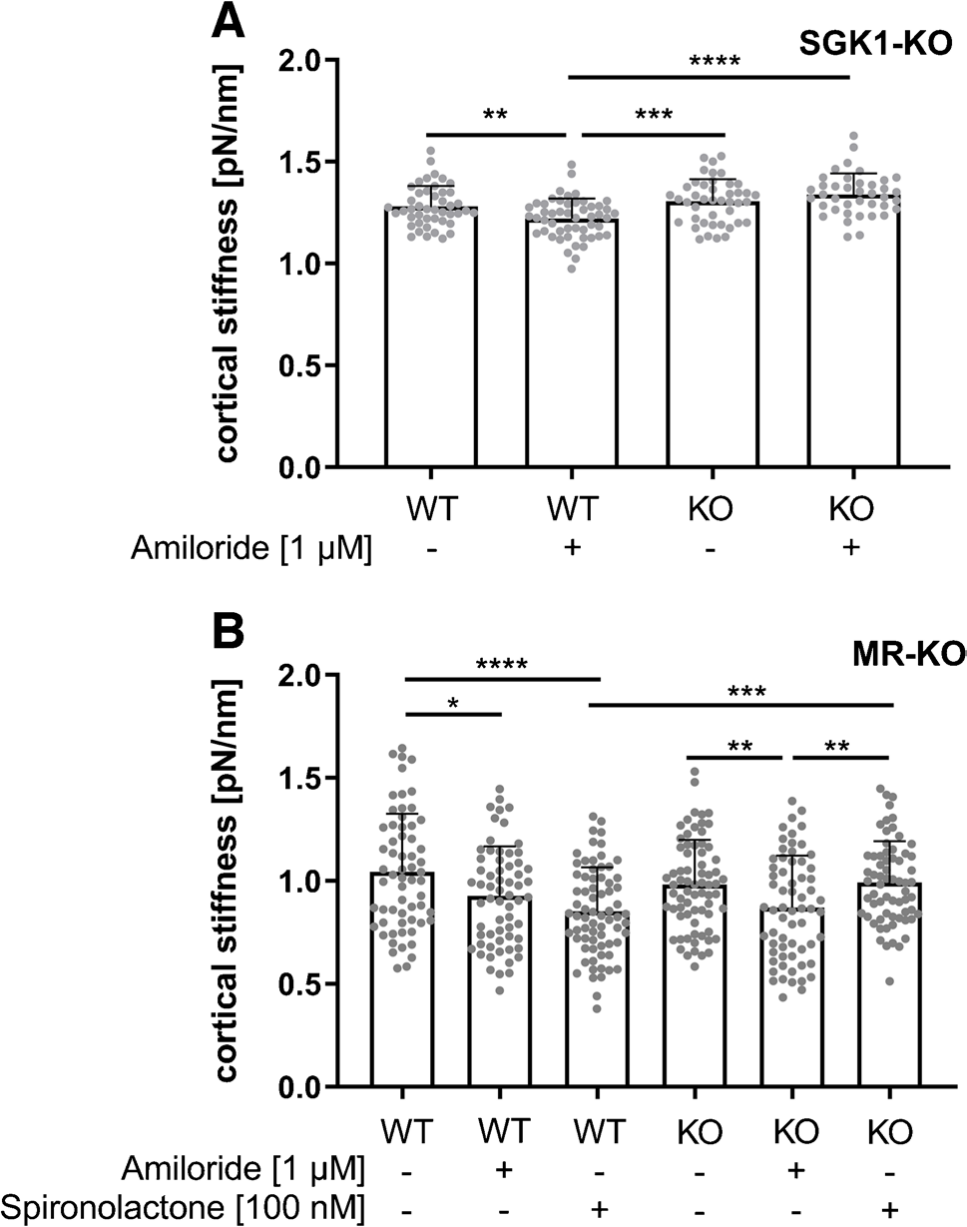
Ex vivo approach demonstrates the involvement of SGK1 and the mineralocorticoid receptor in EnNaC-dependent nanomechanical properties. (**A**) Cortical stiffness was measured on ex vivo EC of aortic patches of WT and SGK1-KO mice. Amiloride treatment (1 µM) led to reduced cortical stiffness in WT cells, whereas this amiloride effect was abolished in SGK1-KO EC (N = 3, n = 39–51; ** *p* < 0.01, **** *p* < 0.0001 vs. control). **(B)** Stiffness of the cortical actin was measured on ex vivo EC of aortic patches of WT and endoMR-KO mice. Direct EnNaC inhibition (amiloride, 1 µM,) as well as MR-antagonist spironolactone (100 nM) led to softening of endothelial cell cortex of WT-mice. Spironolactone has no effect on EC of endoMR-KO aortic patches (*N* = 4, *n* = 62–71, ** *p* < 0.01, *** *p* < 0.001, **** *p* < 0.0001 vs. control)

An ex vivo approach on endothelial cells derived from aortic patches of serum- and glucocorticoid-induced protein kinase1 (SGK1)-KO mice was used to further elucidate the signalling cascades involved in EnNaC regulation. Within wild-type (WT) controls, ex vivo endothelial cells showed reduced cortical stiffness after chronic amiloride treatment (1 µM, 24 h; Fig. 6A; WT 1.28 ± 0.01 pN/nm vs. WT + amiloride 1.22 ± 0.01 pN/nm). In SGK1-KO ex vivo endothelial aortic patches, amiloride failed to alter the endothelial cortical stiffness (Fig. 6A; SGK1-KO 1.30 ± 0.11 pN/nm vs. SGK1-KO + amiloride 1.34 ± 0.10 pN/nm).

Besides the SGK1-Nedd4-2 axis, also, the mineralocorticoid receptor (MR) is involved in the EnNaC-mediated alteration of cortical nanomechanics. In an ex vivo approach with aortic patches of WT vs. endoMR-KO mice, cortical stiffness was measured in ex vivo endothelial cells (Fig. 6B). In WT, chronic inhibition of EnNaC by amiloride led to reduced cortical stiffness (1 µM, 24 h, WT 1.04 ± 0.28 pN/nm vs WT + amiloride 0.92 ± 0.24 pN/nm). Treatment with the MR-antagonist spironolactone (100 nM, 24 h) reduced the cortical stiffness compared to the aldosterone control group (WT + spironolactone 0.85 ± 0.21 pN/nm). In endoMR-KO endothelial cells, functional EnNaC inhibition by amiloride led to reduced cortical stiffness (KO 0.98 ± 0.22 pN/nm vs. KO + amiloride 0.87 ± 0.25 pN/nm), whereas the MR antagonist spironolactone failed to alter the cortical stiffness (KO + spironolactone 0.99 ± 0.20 pN/nm).

## Discussion

The mechanistic basis of how EnNaC determines the stiffness and function of endothelial cells remains to be determined. In the past, it was postulated that it could rely on either the channel’s activity (i.e. Na^+^ influx) or its co-localization with F-actin. Both factors involve changes in the G- to F-actin ratio in the cortical cytoskeleton, leading to increased cortical stiffness and reduced NO release [39, 40]. Recently, the EnNaC membrane abundance and the degree of cortical stiffness could be correlated: The more EnNaC, the stiffer the endothelial cortex [39].

The present study adds details to the underlying mechanisms. We could demonstrate that (i) PI3 K and PKC are involved in rapid aldosterone-induced EnNaC membrane insertion, (ii) the postulated EnNaC-actin interplay relies on small GTPases Rac1 and RhoA, and (iii) Nedd4-2 is involved in the regulation and endocytosis of the channel—possibly via SGK1 signalling.

Notably, the aldosterone-induced impact on the MR axis involves several effectors beyond EnNaC, such as the Na⁺/H⁺ exchanger (NHE3), Na⁺/Cl⁻ cotransporter (NCC), renal outer medullary K⁺ (ROMK) channel, and the Na⁺/K⁺-ATPase [53, 77, 90, 96, 99]. The functional role of endothelial EnNaC in various vascular beds, species, and endothelial cell lines remains a subject of ongoing debate, with both beneficial and detrimental effects being reported [54, 70, 98, 108].

EnNaC has been shown to play a critical role in several cellular mechanisms, including vascular tone regulation, shear stress sensing, and modulation of nitric oxide (NO) production [39, 70, 91]. For instance, in lung capillaries, activation of the α-subunit of EnNaC enhances endothelial barrier function [15]. Further, Tarjus et al. demonstrated that α-subunit contributes to agonist-induced NO production. However, in vessels of mice lacking α-EnNaC, NO production in response to agonists remained intact, likely due to compensatory mechanisms [91]. Lipopolysaccharide (LPS) exposure impaired both acetylcholine- and flow-induced vasodilation in α-EnNaC knockout (KO) mice, correlating with compromised endothelial barrier integrity [87]. In instances where agonist-induced NO production is absent in α-EnNaC KO mice [87, 91], alternative pathways, such as those mediated by endothelium-derived hyperpolarizing factor (EDHF), may be upregulated to compensate for the reduced NO signalling [33].

Given that EnNaC has been identified as a negative regulator of endothelin-1 (ET-1), its inhibition could lift the suppression on ET-1 expression, potentially explaining the flow-mediated vasoconstriction observed in mesenteric arteries following amiloride treatment [67]. Moreover, the responsiveness of EnNaC to amiloride may differ across vascular regions. For example, Martinez-Lemus et al. found that amiloride had no effect on EnNaC expression in aortic endothelium but significantly reduced its levels in mesenteric arteries [61].

In epithelial cells, the aldosterone-induced increase in cell surface ENaC expression and channel open probability is regulated by either trafficking and stabilization of pre-formed ENaC subunits at the apical cell membrane (non-genomic) or through the MR-dependent regulation of ENaC subunit gene transcription (genomic) [18, 30, 32, 34]. Long-term treatment (genomic, 3 day to 3 weeks, in vitro) with the mineralocorticoid receptor antagonist spironolactone mitigates aldosterone-induced EnNaC insertion and polymerization of the cortical actin restores NO bioavailability and therefore prevents the manifestation of the ‘stiff endothelial cell syndrome’ (SECS) [21].

In contrast to the long-term effect of aldosterone stimulation, inducing mainly genomic mechanisms and transcription of aldosterone-induced proteins, this project aims to elucidate the underlying cellular mechanisms and putative time-sensitivity of the rapid aldosterone-induced EnNaC membrane insertion and stiffening of the endothelial surface. In this context, a rapid membrane insertion of endothelial EnNaC due to increased fluid shear stress was shown recently [14], which enables the vascular endothelial cells to effectively react to hemodynamic changes within minutes, independent of kidney-derived mechanisms of blood pressure regulation. This emphasizes the role of EnNaC as an auxiliary controller of vascular tone and blood pressure [91].

A novel finding of the present study is that in endothelial cells, EnNaC membrane insertion after treatment with 1 nM aldosterone occurs rapidly within 7 min, which is independent of the MR but sensitive to Brefeldin A, indicating that EnNaC molecules are inserted non-genomically from intracellular vesicular pools. This rapid aldosterone-induced EnNaC membrane insertion is accompanied by actin polymerization and subsequent mechanical stiffening of the endothelial cortex. We could demonstrate that the rapid EnNaC membrane insertion is mediated mainly by PI3 K and PKC signalling pathways. In contrast, ENaC regulation in other cell types thereby seems to differ from endothelial regulation. Stockand et al. showed in renal A6 cell line by using the PKC activator phorbol 12-myristate 13-acetate an decrease in sodium reabsorption and different effects of PKC activation and/or inhibition dependent on ENaC subunits [88]. ENaC beta-subunit seems to be necessary for PKC-induced inhibition of renal ENaC [74]. Also, possible side-effects of PCK inhibitor Chelerythrine on MAPK pathway should be concerned.

Functional inhibition of EnNaC with amiloride or benzamil led to significant softening of the cell cortex within minutes, which was accompanied by actin depolymerization. Here, we could show that this process is mediated by clathrin-dependent endocytosis of the channel.

Our findings led to the further conclusion that EnNaC function, i.e. Na^+^ influx into the cell, has an impact on the polymerization level of actin, the G- to F-actin ratio, and thus the mechanical surface properties of endothelial cells. This might probably occur via increasing intracellular Na^+^ concentrations, which facilitate the exchange of Na^+^ with Ca^2+^ through the Na^+^/Ca^2+^ exchanger (NCX) and further activate downstream signalling pathways [12, 73, 78]. The physiological relevance of NCX in the reverse mode (Na^+^ export and Ca^2+^ entry) has been reported in a different cell types, including endothelial cell [2, 72, 93].

Recently, an elevated ENaC membrane channel density has been linked to vesicle trafficking processes via an intact cortical cytoskeleton and coupled to the activation of small GTPases in epithelial cells [44–46, 62]. The involvement of small GTPases in rapid EnNaC membrane insertion and endothelial stiffening could also be demonstrated in the present study. In particular, in endothelial cells, Rac1, RhoA, and the Arp2/3 complex are responsible for this. We demonstrated that inhibition of these signalling pathways reduced both the presence of EnNaC in the plasma membrane of endothelial cells and the mechanical stiffness of the cortex. Inhibition of these pathways also results in depolymerization of cortical actin. This clearly highlights the interdependence between the cortical cytoskeleton, EnNaC membrane insertion, and the resulting mechanical properties of the endothelial surface. This agrees with data showing that the nanomechanical properties of endothelial cells including augmented cortical stiffness and cytoskeleton remodelling within *en face* aorta are dependent on the Rac1 pathway [66]. In vitro manipulation of the cortical stiffness using Jasplakinolide and Cytochalasin D demonstrates that EnNaC membrane abundance correlates with the stiffness degree of the cortex.

Besides membrane insertion, EnNaC may be regulated by removal from the cell membrane. A prominent regulator of ENaC membrane trafficking in epithelial cells and heterologous expression systems such as *Xenopus laevis* oocytes is the aldosterone-induced serum and glucocorticoid-induced kinase 1 (SGK1) [16, 58, 104]. The C-termini of all three ENaC subunits contain proline-rich PPPxY (PY) motifs, and phosphorylation of Nedd4-2 by SGK1 reduces the affinity of the PPPxY-motif to Nedd4-2, which prevents channel retrieval from the cell surface, thereby increasing channel surface abundance [6, 38, 84, 85]. This mechanism seems to include clathrin-mediated endocytosis [83, 97, 102]. Our current observations indicate that Nedd4-2 and SGK1 also have functional roles in EnNaC trafficking, as modulation of one of the two factors alters cellular stiffness. The increased stiffness in endothelial cells after Nedd4-2 silencing correlates with a higher EnNaC membrane abundance, as EnNaC inhibition is able to ameliorate the increased stiffness. In turn, endothelial stiffness was insensitive to amiloride in SGK1-deficiency, suggesting a dysfunctional signalling pathway. However, endothelial stiffness was not altered in SGK1 deficiency under control conditions, indicating that putative effects of SGK1 deficiency on endothelial stiffness might be compensated by other pathways or mechanisms. This is in agreement with the observations that SGK1 deficient mice show no profound phenotype without any stimulation, and no alterations in blood pressure [59]. In accordance, endothelial stiffness was also not altered in MR-deficient endothelial cells. However, amiloride still was able to induce EC softening, while spironolactone did not induce significant alterations. These observations support a contribution of MR, SGK1, and Nedd4-2 in EnNaC membrane regulation.

In conclusion, we provide novel data on the regulation of EnNaC in vascular endothelial cells. In particular, the interplay between EnNaC and the cortical cytoskeleton through small GTPases highlights the importance of the structure–function relationship of EnNaC and its specific role in the vasculature. Moreover, we could show an involvement of SGK1 and Nedd4-2 in the regulation of the membrane insertion/retrieval of the channel.

We postulate that via EnNaC membrane abundance and its connection to the cortical cytoskeleton, endothelial nanomechanics can be manipulated and a physiological switch between a ‘soft’ and ‘stiff’ cell surface and thus proper cell behaviour is ensured. Under pathophysiological and inflammatory conditions, this balanced mechanism could be disrupted, which could then promote SECS, endothelial, and vascular dysfunction.

## Supplementary Information

Below is the link to the electronic supplementary material.

ESM 1 (PNG 48.4 KB)

ESM 2 (PNG 161 KB)

ESM 3 (PNG 1.14 MB)

ESM 4 (PNG 782 KB)

## Data Availability

No datasets were generated or analysed during the current study.
