# Supplementary figures and images for: SECS, drugs, and Rac1&Rho: regulation of EnNaC in vascular endothelial cells
Source: Pflugers Arch. 2025 May 22;477(7):977–92. doi: 10.1007/s00424-025-03093-5 (PMC12152027; doi:10.1007/s00424-025-03093-5)

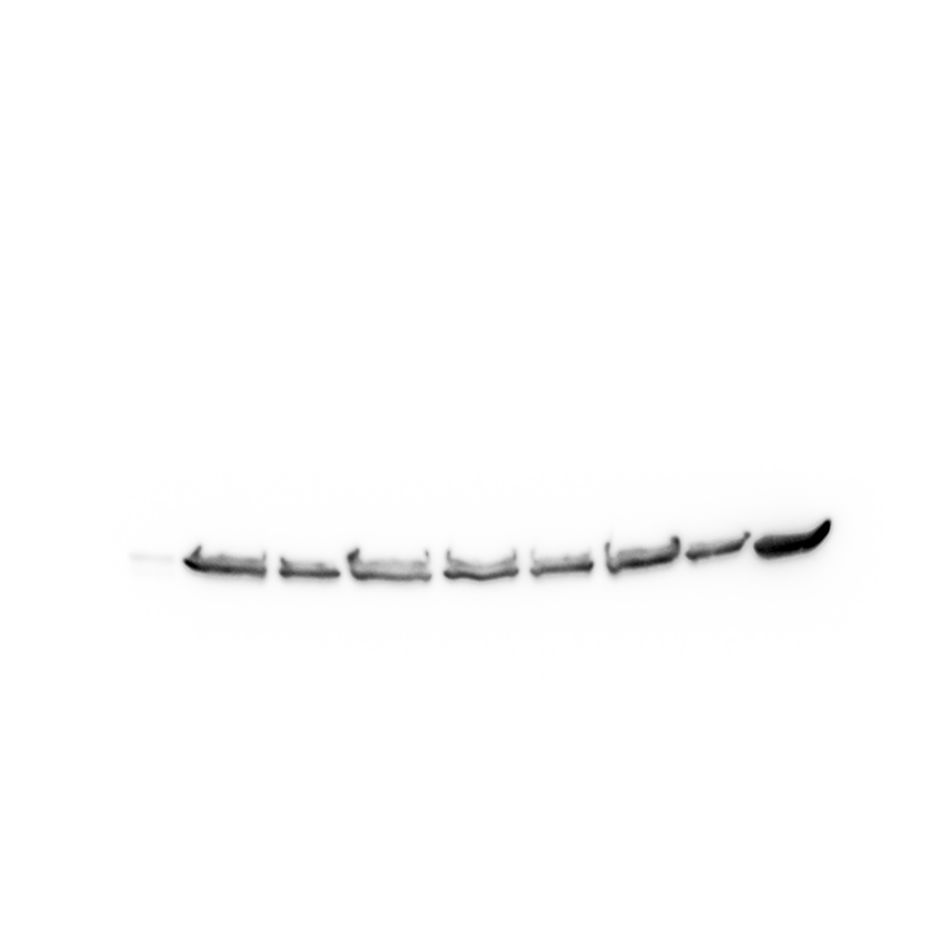

Supplement: Supplementary file 1 — (PNG 48.4 KB) [file 424_2025_3093_MOESM1_ESM.png]

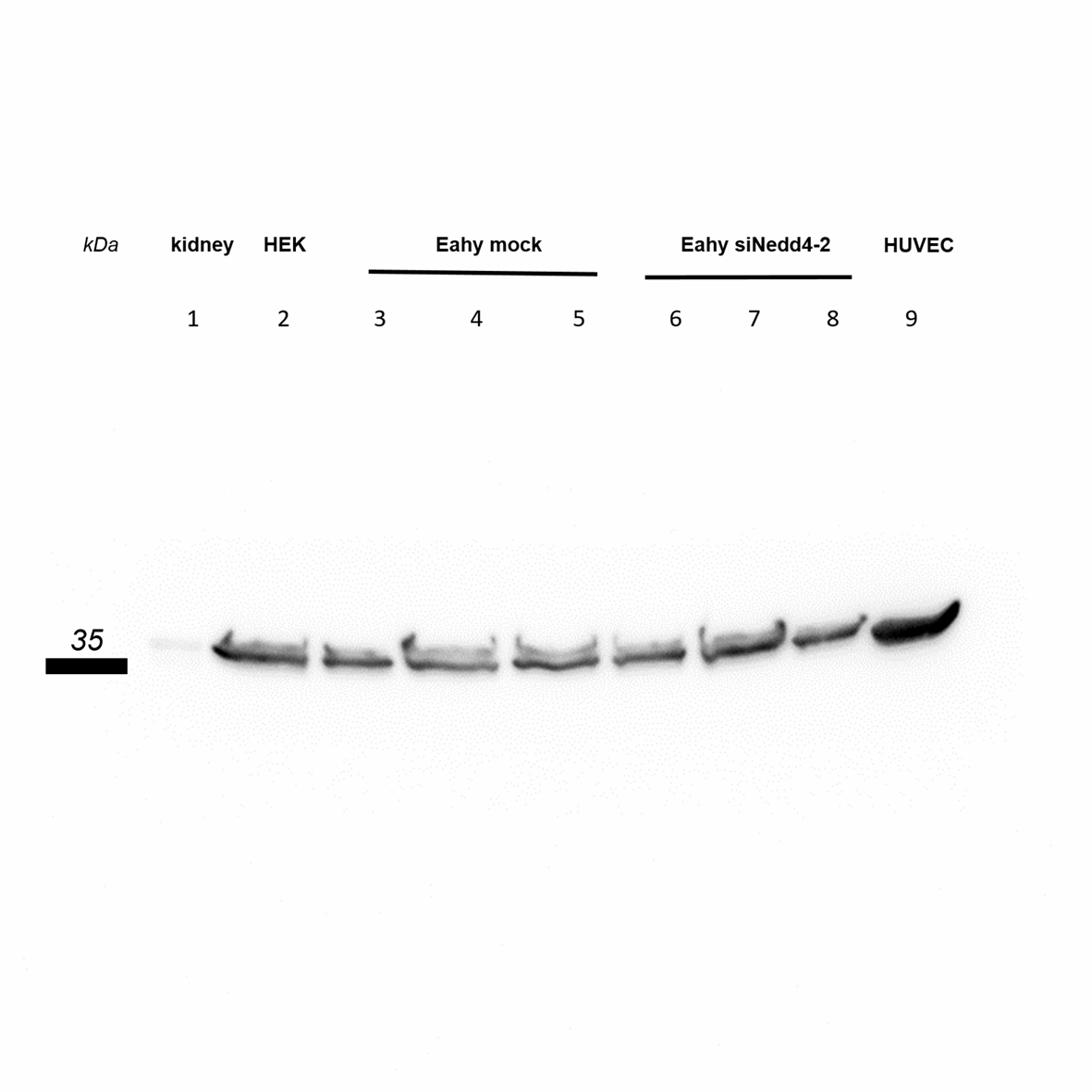

Supplement: Supplementary file 2 — (PNG 161 KB) [file 424_2025_3093_MOESM2_ESM.png]

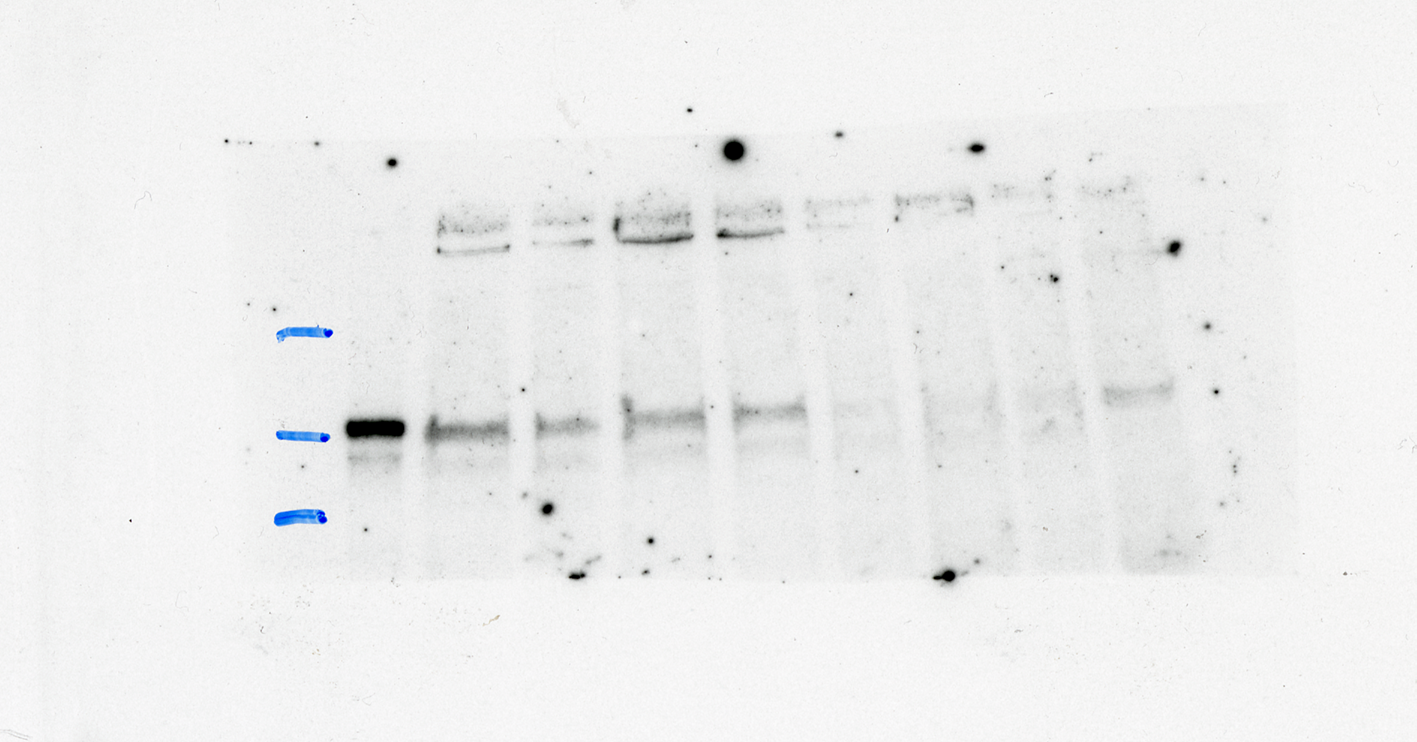

Supplement: Supplementary file 3 — (PNG 1.14 MB) [file 424_2025_3093_MOESM3_ESM.png]

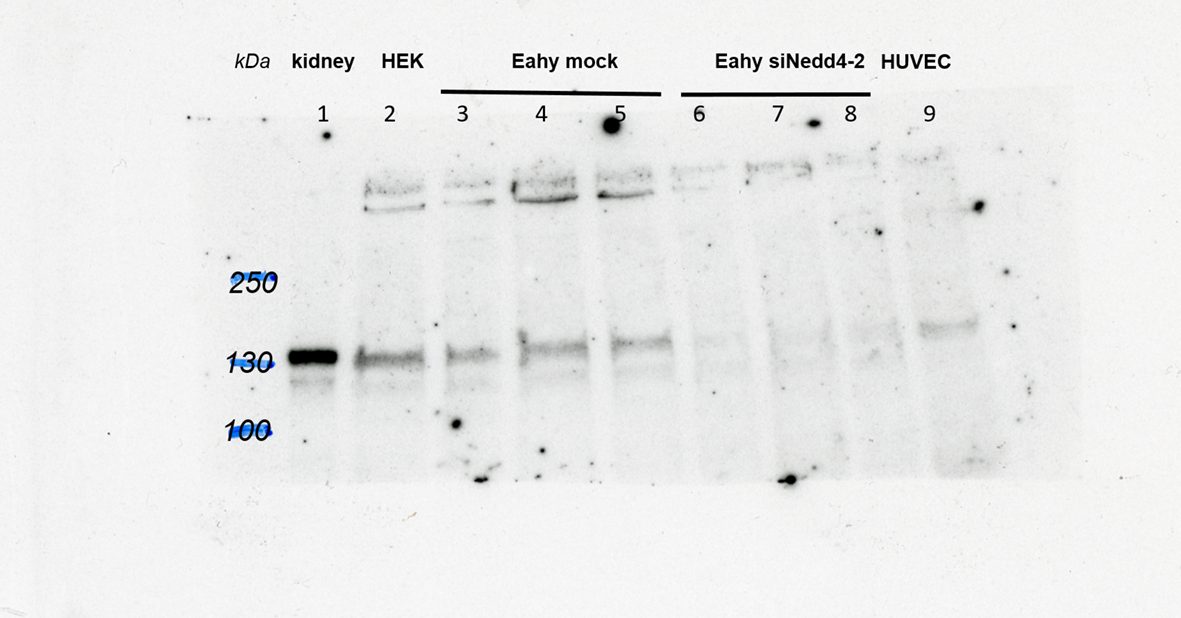

Supplement: Supplementary file 4 — (PNG 782 KB) [file 424_2025_3093_MOESM4_ESM.png]
